# Molecular Modeling Study of c-KIT/PDGFRα Dual Inhibitors for the Treatment of Gastrointestinal Stromal Tumors

**DOI:** 10.3390/ijms21218232

**Published:** 2020-11-03

**Authors:** Seketoulie Keretsu, Suparna Ghosh, Seung Joo Cho

**Affiliations:** 1 Department of Biomedical Sciences, College of Medicine, Chosun University, Gwangju 501-759, Korea; keretsu@chosun.kr (S.K.); s.ghosh@chosun.kr (S.G.); 2 Department of Cellular Molecular Medicine, College of Medicine, Chosun University, Gwangju 501-759, Korea

**Keywords:** c-KIT, PDGFRα, molecular dynamics simulation, free energy calculation, CoMFA, CoMSIA, 3D-QSAR

## Abstract

Gastrointestinal stromal tumors (GISTs) are the most common Mesenchymal Neoplasm of the gastrointestinal tract. The tumorigenesis of GISTs has been associated with the gain-of-function mutation and abnormal activation of the stem cell factor receptor (c-KIT) and platelet-derived growth factor receptor alpha (PDGFRα) kinases. Hence, inhibitors that target c-KIT and PDGFRα could be a therapeutic option for the treatment of GISTs. The available approved c-KIT/PDGFRα inhibitors possessed low efficacy with off-target effects, which necessitated the development of potent inhibitors. We performed computational studies of 48 pyrazolopyridine derivatives that showed inhibitory activity against c-KIT and PDGFRα to study the structural properties important for inhibition of both the kinases. The derivative of phenylurea, which has high activities for both c-KIT (pIC_50_ = 8.6) and PDGFRα (pIC_50_ = 8.1), was used as the representative compound for the dataset. Molecular docking and molecular dynamics simulation (100 ns) of compound **14** was performed. Compound **14** showed the formation of hydrogen bonding with Cys673, Glu640, and Asp810 in c-KIT, and Cys677, Glu644, and Asp836 in PDGFRα. The results also suggested that Thr670/T674 substitution in c-KIT/PDGFRα induced conformational changes at the binding site of the receptors. Three-dimensional quantitative structure–activity relationship (3D-QSAR) models were developed based on the inhibitors. Contour map analysis showed that electropositive and bulky substituents at the para-position and the meta-position of the benzyl ring of compound **14** was favorable and may increase the inhibitory activity against both c-KIT and PDGFRα. Analysis of the results suggested that having bulky and hydrophobic substituents that extend into the hydrophobic pocket of the binding site increases the activity for both c-KIT and PDGFRα. Based on the contour map analysis, 50 compounds were designed, and the activities were predicted. An evaluation of binding free energy showed that eight of the designed compounds have potential binding affinity with c-KIT/PDGFRα. Absorption, distribution, metabolism, excretion and toxicity (ADMET) and synthetic feasibility tests showed that the designed compounds have reasonable pharmaceutical properties and synthetic feasibility. Further experimental study of the designed compounds is recommended. The structural information from this study could provide useful insight into the future development of c-KIT and PDGFRα inhibitors.

## 1. Introduction

Gastrointestinal stromal tumors (GISTs) are the most common mesenchymal tumors of the gastrointestinal tract that arise from interstitial cells of Cajal (ICC) or from stem cells that differentiate toward ICCs [1,2]. GISTs are commonly originated in the stomach (70%), with rare cases of it observed in the small intestine (20%) or esophagus (10%) [3]. It has an incidence rate of 14.5 per million per year [4].

Stem cell factor receptor (c-KIT) [5] and platelet-derived growth factor receptor alpha (PDGFRα) [6] kinases are members of Type 3 transmembrane receptor protein–tyrosine kinase (RPTK) family and play important roles in various cellular signaling processes. Structurally, the member of the RPTK subfamily consists of five extracellular immunoglobulin (Ig) domains, one transmembrane domain, one juxtamembrane helix, and one cytoplasmic kinase domain [7]. The c-KIT kinases are primarily expressed on the hematopoietic stem cell surface and bind to the stem cell factor at the extracellular Ig domain. The binding of the stem cell factor to c-KIT leads to the dimerization of the kinase domains and the phosphorylation of specific tyrosine residues in the juxtamembrane regions, which in turn activates downstream signaling cascades that mediate cell survival, proliferation, and differentiation [8,9]. The PDGFRα kinases are expressed on the surface of several cell types and bind to the platelet-derived growth factor. The binding of the platelet-derived growth factor leads to kinase domain dimerization and activation. PDGFRα plays an important role in the regulation of embryonic development, cell proliferation, and cell survival [10,11]. Experimental studies have shown that the tumorigenesis of GISTs is associated with the gain-of-function mutation in c-KIT and PDGFRα [12,13,14]. Rammohan et al. (2013) reported that positive c-KIT expression was observed in approximately 90% of GISTs cases [15]. Mutations that lead to constitutive PDGFRα activation have been found in approximately 10% of GISTs cases [16]. As a result of the role of the c-KIT and PDGFRα in the development and progression of GISTs, these kinases are considered to be promising therapeutic targets for the treatment of GISTs.

So far, three non-selective inhibitors, namely, imatinib, sunitinib, and regorafenib have been approved by the Food and Drug Administration (FDA) for the treatment of GISTs [17]. Imatinib is a non-selective inhibitor of c-KIT, PDGFRα, and ABL (Abelson) kinases and has been approved for use as a first-line treatment of GISTs. However, one-half of the responding GISTs patients gain Imatinib resistance within 2 years of treatment via mutation at the T670 residue of c-KIT [18]. The result is more optimistic in the case of PDGFRα with only 5–7% of cases with PDGFRα mutation showing resistance to imatinib [19]. Sunitinib is another non-selective inhibitor of c-KIT, PDGFRα, vascular endothelial growth factor receptor (VEGFR), and Fms like tyrosine kinase 3 (FLT3) that has been approved for the second-line treatment of GISTs [20]. Regorafenib is also a non-selective multi-kinase inhibitor with activity against c-KIT, PDGFRα, VEGFR, rapidly accelerated fibrosarcoma 1 (RAF1), rearranged during transfection (RET), and fibroblast growth factor receptor (FGFR) [21,22]. However, an efficacy and safety study of Regorafenib in patients with advanced GISTs has shown that 15% of the patients experience an exacerbation of cancer-related symptoms [23]. In addition to the approved drugs, several other c-KIT/PDGFRα inhibitors such as dovitinib [24], masitinib [25], crenolanib [26], and ripretinib [27] are also under investigation for the treatment of GISTs [28]. Although progress has been made in the treatment of GISTs, current therapeutic options have various drawbacks such as low efficacy, clinical resistance, and side effects due to the non-selective property of the existing drugs.

Computer-aided drug discovery (CADD) methods have become popular in the drug discovery process and have been widely used in several drug discovery studies [29,30,31,32]. Taking advantage of the CADD techniques, our research group has performed several computational studies particularly in the area of kinase inhibitors [33,34,35,36]. Given the role of both c-KIT and PDGFRα in the tumorigenesis of GISTs, the computational study of the dual inhibitory mechanism of these kinases could provide valuable insight into developing more effective drugs against GISTs. In this spirit, we have performed the computational studies of a series of potent and selective pyrazolopyridine inhibitors to explore the structural features important for the dual inhibition of c-KIT/PDGFRα. Molecular docking and molecular dynamics (MD) simulation were performed to study the inhibitor–protein binding interactions. Comparative molecular field analysis (CoMFA) [37] and comparative molecular similarity indices analysis (CoMSIA) [38] models were developed, and the contour maps were analyzed to explore the important structural features. Binding energy evaluation was carried out to predict the binding affinity of the compounds.

## 2. Results

The X-ray crystal structures of imatinib in complex with the inactive form of c-KIT (PDB ID 1T46) and PDGFRα (PDB ID 6JOL) were collected from the protein databank (www.rcsb.org). Imatinib showed a pIC_50_ value of 7.4 and 8.3 for c-KIT and PDGFRα respectively and was used as a reference compound [39]. The dataset compound **14**, which showed high pIC_50_ values of 8.6 for c-KIT and 8.1 for PDGFRα, was used as a representative compound for the dataset. The dataset compounds and their log activity values are shown in Table 1.

### 2.1. Molecular Docking

Molecular docking was performed using Autodock 4.2. Validation of the docking procedure was performed by docking the crystal ligand (imatinib) into the receptors. The overlap between the docked pose and the corresponding X-ray structure inside c-KIT and PDGFRα are shown in Figure S1 (Supplementary Material). Docking results showed that imatinib formed H-bond interactions with Cys673, Thr670, Glu640, and Ile769 in c-KIT and Cys677, Thr836, Glu644, Val815, and His816 in PDGFRα.

Docking of the compound **14** with c-KIT showed H-bond interactions with Cys673 at the hinge region, Glu640 at the αC-helix, and Asp810 at the DFG motif of the activation loop. The binding interaction of pyrazolopyridine of compound **14** with hinge residue Cys673 was analogous to the interaction of the pyridine of imatinib with Cys673 observed in the X-ray structure (PDB ID 1T46). This interaction with the hinge region was crucial for anchoring the ligand at the binding site. Compound **14** showed H-bond interaction with PDGFRα residues Cys677 (hinge), Glu644 (αC-helix), and Asp836 (DFG motif). The results suggested that compound **14** was bound to c-KIT and PDGFRα in a similar binding pattern. The docked poses of compound **14** inside the receptors are shown in Figure S1 (Supplementary Material).

### 2.2. Molecular Dynamics Simulation

**Figure 1:**
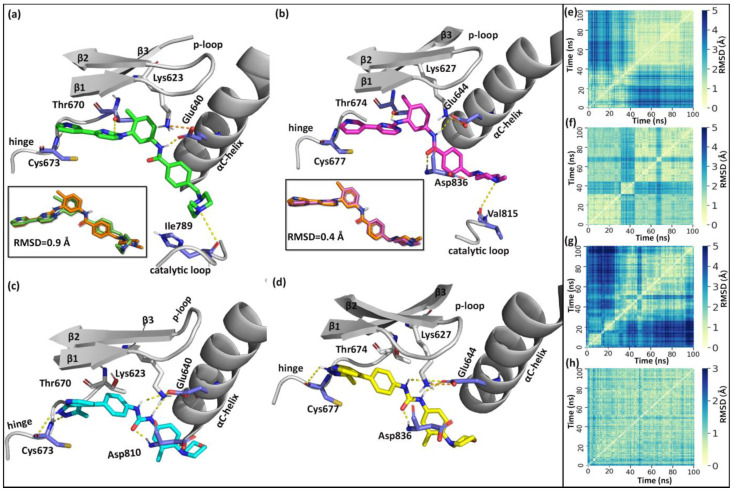
H-bond interactions of imatinib and compound **14** with c-KIT and PDGFRα from the molecular dynamics (MD) simulations. Proteins were represented in grey color cartoon representation. H-bond interactions are represented by yellow dotted lines and residues forming H-bonds are shown in a purple color. (**a**) Binding interactions between imatinib and c-KIT. The overlap between the crystal ligand pose (salmon) and the MD binding pose (green) of imatinib at the binding site (root mean square deviation (RMSD) = 0.9 Å). (**b**) Binding interactions between imatinib and PDGFRα. The overlap between the crystal ligand pose (salmon) and the MD binding pose (magenta) of imatinib at the binding site (RMSD = 0.4 Å). (**c**) Binding interactions between compound **14** (cyan)_ and c-KIT. (**d**) Binding interactions between compound **14** and PDGFRα. The pairwise RMSD plots of the ligands from the MD simulation of (**e**) imatinib and c-KIT (**f**) imatinib and PDGFRα (**g**) compound **14** and c-KIT (**h**) compound **14** and PDGFRα. The protein–ligand complexes were first aligned by least square fitting the protein backbone atoms. The ligand RMSD was calculated based on the ligand heavy atoms. The pairwise RMSD plot shows the ligand RMSD of each frame in the trajectory.

Classical MD simulations of imatinib and compound **14** with c-KIT and PDGFRα were performed for 100 ns using Gromacs. The interactions of imatinib and compound **14** with the receptors and the pairwise root mean square deviation (RMSD) of the ligands from the MD trajectories are shown in Figure 1. During the simulation of the imatinib–c-KIT complex, the αC-helix and the activation loop formed a narrow pocket around the binding site (Figure 1a). This allowed the Glu640 from the αC-helix to form a stable salt bridge with Lys623 of β3 and one H-bond with the amide linker of imatinib. Imatinib also formed a stable H-bond with Cys673 at the hinge and Thr670 of the gate-keeper residue. The methyl piperazine moiety of imatinib also formed a weak H-bond with the Ile789 of the catalytic loop. The overlap between the crystal ligand and the MD binding pose showed an RMSD value of 0.9 Å.

In PDGFRα (Figure 1b), imatinib formed an H-bond interaction with Cys677 and Thr674 at the hinge region. The carbonyl linker between the two benzyl rings of imatinib also formed an H-bond interaction with the Asp836 at the DFG motif. Additionally, a weak H-bond interaction was also observed between the methyl piperazine of imatinib and the catalytic loop residue Val815. These interactions were also observed in the X-ray structure of the imatinib-PDGFRα (PDB ID 6JOL). The overlap of the crystal ligand and the MD binding pose showed an RMSD value of 0.4 Å.

In the compound **14-c-KIT** complex simulation, the pyrazolopyridine of compound **14** occupied the pocket close to the hinge region and formed H-bond interactions with Cys673. Additionally, compound **14** formed H-bond interactions with Glu640 and Asp810 from the αC-helix and the DFG-motif, respectively. The morpholine moiety of compound **14** extended into the hydrophobic pocket formed by residues Ile571, Val643, Leu647, Phe782, Leu783, Cys788, and Ile789 from the αC-helix and the catalytic domain (Figure S2, Supplementary Material). The binding pose of compound **14** inside c-KIT is shown in Figure 1c. In PDGFRα, compound **14** formed H-bond interactions with Cys677 (hinge), Glu644 (αC-helix), and Asp836 (DFG motif). The binding pose of compound **14** inside PDGFRα is given in Figure 1d. These results indicated that compound **14** formed interactions with c-KIT and PDGFRα in a similar pattern.

The interactions observed in the MD simulations were also observed in the X-ray crystal structures. The root mean square deviation (RMSD) between the MD pose of imatinib and the corresponding X-ray crystal ligand was 0.9 Å in c-KIT and 0.4 Å in PDGFRα, suggesting that the MD simulations were able to replicate the binding interactions reasonably. The interaction of compound **14** with Cys673 at the hinge region was similar with the interactions observed in the X-ray crystal structure of the imatinib–c-KIT complex (PDB ID 1T46). The interactions of compound **14** with Glu640, Lys623, and Asp810 were similar with the interactions seen in the crystal structure of DP2976-c-KIT (PDB ID 6MOB). (Page 8, Line 149-155). Similarly, the imatinib–PDGFRα interactions observed in the MD simulation were also similar with the interactions observed in the crystal structures of imatinib–PDGFRα (PDB ID 6JOL).

### 2.3. Evaluation of Binding Energy

The binding energy (BE) of imatinib and compound **14** with c-KIT and PDGFRα was evaluated using the g_mmpbsa package. The contributions of van der Waals, electrostatic, polar, and non-polar solvation energy terms to the total BE of the protein–ligand complexes were given in Table 2. The total BE of imatinib–c-KIT and imatinib–PDGFRα were −105 kJ/mol and −104 kJ/mol, respectively. The van der Waals and electrostatic energy terms made the major contributions to the total BE. In imatinib–c-KIT binding, the van der Waals and electrostatic energies contributed −236 kJ/mol and −70 kJ/mol to the total BE, respectively. In imatinib–PDGFRα, the van der Waals and electrostatic energy contributions were −244 kJ/mol and −58 kJ/mol, respectively. The total BE values of compound **14-c-KIT** and compound **14-PDGFRa** were −120 kJ/mol and −117 kJ/mol, respectively. In the compound **14**-**c-KIT** interaction, the van der Waals and electrostatic energies contributed −257 kJ/mol and −57 kJ/mol to the total BE, respectively. In compound **14-PDGFRα** interaction, the van der Waals energy contribution was −251 kJ/mol, and the electrostatic energy contribution was −55 kJ/mol.

The residues that made a high contribution to the total BE in compound **14–c-KIT** interaction were compared with the corresponding residues in imatinib–c-KIT interaction in Table 3. The results indicated that the hydrophobic residues Val603, Leu644, Val654, Cys809, and Phe811 individually contributed more than −5 kJ/mol to the total BE in compound **14–c-KIT** interaction. In imatinib–c-KIT, the hydrophobic residues Leu644, Val654, Tyr672, and Cys809 individually contributed more than −5 kJ/mol to the total BE. The hydrophobic residues Val607, Met648, Val658, Leu825, and Cys835 individually contributed more than −5 kJ/mol to the total BE in both imatinib–PDGFRα and comound **14–PDGFRα** interactions. The polar residue Asp836 contributed −5.1 kJ/mol in compound **14**–**PDGFRα** interaction and 1.8 kJ/mol in imatinib–PDGFRα interaction. Similarly, the corresponding c-KIT residue Asp810 contributed −2.1 kJ/mol in compound **14–c-KIT** interaction and 9.3 kJ/mol in imatinib–c-KIT interaction. The high-energy contribution of Asp836/Asp810 in the interactions with compound **14** may be attributed to the H-bond interaction between the carbonyl oxygen of compound **14** with Asp836/Asp810 in c-KIT/PDGFRα. The PDGFRα residue Phe837 also contributed −4.8 kJ/mol and −7.1 kJ/mol in the interactions with compound **14** and imatinib, respectively. The corresponding c-KIT residue Phe811 also contributed −6.8 kJ/mol and −4 kJ/mol in interactions with compound **14** and imatinib respectively through hydrophobic interactions. These hydrophobic interactions with Phe811/Phe837 of the DFG motive were possible as a result of the Asp-Phe-Gly/DFG in conformation of the inactive form of c-KIT and PDGFRα [5].

### 2.4. D-QSAR

The 48 pyrazolopyridine derivatives and their activity values were used to perform the 3D-QSAR study. The specific activity values of compounds **3**, **4**, **24**, **25**, **34**, and **38** for both receptors were not available and were excluded from the 3D-QSAR study. The compounds were randomly separated into a training set and a test set of 30 compounds and 12 compounds respectively.

**Figure 2:**
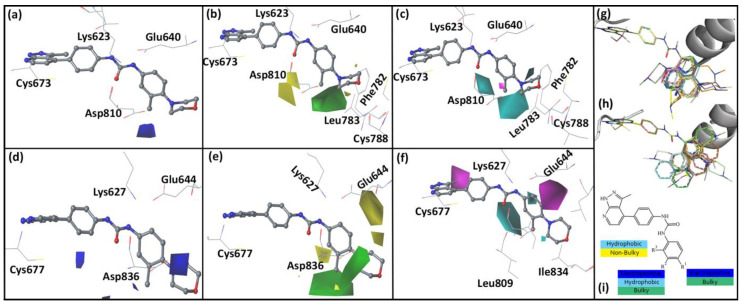
Contour maps generated based on the CoMFA and CoMSIA models for c-KIT and PDGFRα with compound **14** used as a reference. Blue and red contours indicate electropositive and electronegative substituents’ favorable regions, respectively. Green and yellow contours indicate steric bulk substituents’ favorable and unfavorable regions, respectively. Cyan and purple colors contours represent hydrophobic favorable and unfavorable regions. (**a**) Electrostatic contour map for the c-KIT CoMFA model. (**b**) Steric contour map for the c-KIT CoMFA model (**c**) Hydrophobic contour map for the c-KIT CoMSIA model. (**d**) Electrostatic contour map for the PDGFRα CoMFA model. (**e**) Steric contour map for the PDGFRα CoMFA model. (**f**) Hydrophobic contour map for the PDGFRα CoMSIA model. Alignments used for the development of the 3D-QSAR models. (**g**) Alignment of the compounds inside c-KIT. (**h**) Alignment of the compounds inside PDGFRα. (**i**) Scheme developed based on the 3D-QSAR models for designing new compounds.

In the c-KIT CoMFA and CoMSIA models, the binding pose of compound **14** from the MD simulation with c-KIT was used as a template for the alignment of the compounds. The aligned compounds are shown in Figure 2g. The developed CoMFA model showed a crossvalidated *q^2^* value of 0.63 and an optimal number of components (ONC) value of 6. In the non-validated analysis, the model showed an *r^2^* value of 0.98 and SEE value of 0.2, suggesting that the model has a reasonable predictive ability. The CoMSIA model based on the hydrophobic (H) and steric (S) descriptors gave relatively higher statistical results. Hence, this model was selected for further analysis. The selected CoMSIA model exhibited *q^2^* and ONC values of 0.6 and 5, respectively. In the non-crossvalidated analysis, the CoMSIA model showed *r^2^* and standard error of estimation (SEE) values of 0.9 and 0.46. The statistical results of the c-KIT CoMFA and CoMSIA models are shown in Table 4.

In the PDGFRα CoMFA and CoMSIA models, the compounds were aligned based on the binding pose of compound **14** from the MD simulation with PDGFRα. The aligned compounds are shown in Figure 2h. The PDGFRα CoMFA model showed a *q*^2^ value of 0.61 and an ONC value of 6. In the non-validated analysis, the model showed *r*^2^ and SEE values of 0.98 and 0.12, respectively. The PDGFRα CoMSIA model was developed based on the HS descriptors. The PDGFRα CoMSIA model showed *q*^2^ and ONC values of 0.62 and 3, respectively. The non-crossvalidated *r*^2^ and SEE values were 0.81 and 0.39, respectively. The statistical results of the PDGFRα CoMFA and CoMSIA models are shown in Table 4.

Internal and external validation of the derived 3D-QSAR models were performed using bootstrapping (BS) and external *r*^2^*_pred_* analysis. The c-KIT CoMFA model showed BS-*r*^2^ and BS-*SD* values of 0.98 and 0.15, respectively. The c-KIT CoMSIA (SH) model showed a BS-*r*^2^ value of 0.94 and a BS-*SD* value of 0.32. The BS analysis suggested that the c-KIT CoMFA and CoMSIA models have reasonable robustness. The PDGFRα CoMFA model showed a BS-*r*^2^ value of 0.98 and a BS-*SD* value of 0.1. The BS-*r*^2^ and BS-*SD* values for the CoMSIA model were 0.97 and 0.14, respectively. These results suggested that the derived CoMFA and CoMSIA models have reasonable robustness. In the external validation, c-KIT CoMFA and CoMSIA models showed *r*^2^*_pred_* values of 0.59 and 0.58, respectively. The PDGFRα CoMFA and CoMSIA models showed *r*^2^*_pred_* values of 0.56 and 0.59, respectively. The external validation results suggested that the derived models have reasonable predictive ability against an external dataset. The predicted activity values of the compounds for c-KIT and PDGFRα are given in Tables S1 and S2 (Supplementary Material). The scatter plots between the predicted and experimental activity values are given in Figure S3 (Supplementary Material).

### 2.5. Analysis of Contour Map

In the CoMFA and CoMSIA contour maps, compound **14** was used as a reference. The contour maps are shown in Figure 2. In the electrostatic contour map, the red contours represent favorable electronegative substitution for higher activity, whereas the blue contours represent electropositive substitution. The green color in the steric contour map represent regions favorable to bulky substituents for higher activity, whereas yellow contours represent non-bulky substituent favorable regions. In the hydrophobic contour map, cyan contours represent hydrophobic substituent favorable regions for higher activity, whereas purple contours represent hydrophobic substituent unfavorable regions.

In the c-KIT CoMFA electrostatic contour map (Figure 2a), a blue contour was observed near the meta position of the methylbenzene ring suggesting that electropositive substituents were favored at that position. In the steric contour map (Figure 2b), a green contour was observed near the meta position. The yellow contour near the ortho position of methylbenzene indicated that bulky substituents were not favored in that position. Bulky substituents at the ortho position of methylbenzene could lead to a steric clash with binding site residues. In the CoMSIA hydrophobic contour map (Figure 2c), a cyan contour was seen near the para position and meta position of methylbenzene, suggesting that hydrophobic substituents were favorable in these regions. A CoMSIA steric contour map was similar to that of the CoMFA steric contour map and was not included in the analysis.

In the PDGFRα CoMFA electrostatic contour map (Figure 2d), a blue contour was observed near the benzene and the para position of the methylbenzene, suggesting that electropositive substituents were favored in these regions. In the steric contour map (Figure 2e), the green contour was observed near the meta-position of methylbenzene, suggesting that bulky substituents were favored in that region. The yellow contour near the ortho position suggested that bulky substituents were not favored in that region and could lead to decreased activity for both c-KIT and PDGFRα. In the CoMSIA hydrophobic contour map (Figure 2f), a cyan contour was observed near the ortho position of the methylbenzene, and purple contours were observed near the pyrazolopyridine and the meta position of the methylbenzene, suggesting that hydrophobic substituents are not favored in these regions. 

### 2.6. Designed Compounds

Based on the 3D-QSAR contour maps, a design scheme was developed, as shown in Figure 2g. Following the scheme, 50 compounds were designed, and the activity values for c-KIT and PDGFRα were predicted using the derived CoMSIA (SH) models. Based on the predicted pIC_50_ values, eight compounds that showed higher activity values than compound **14** were selected for further evaluations. The predicted activity values of the designed compounds for both the receptors are given in Table 5.

**Figure 3:**
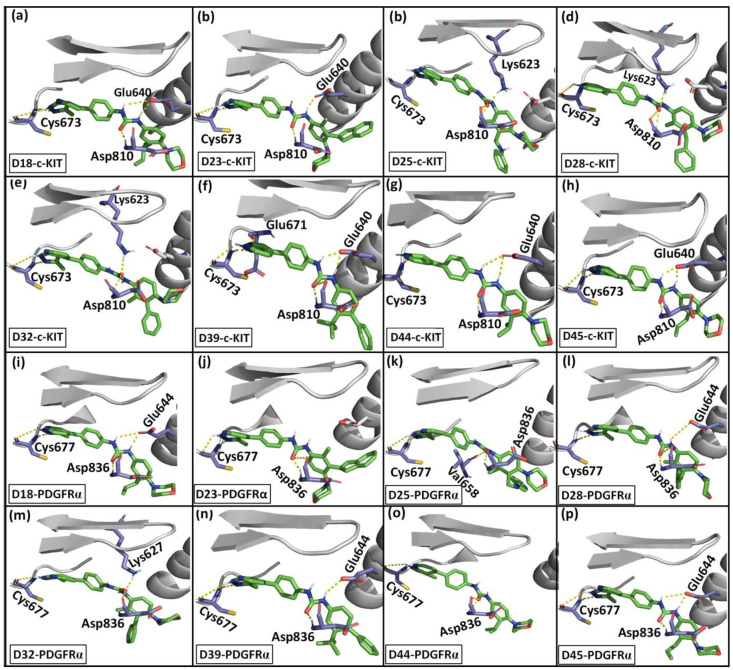
H-bond interactions of the designed compounds with c-KIT and PDGFRα from the MD simulations. Proteins are represented in grey color cartoon representation. Designed compounds are shown in green stick representation. H-bond interactions are represented by yellow dotted lines, and residues forming H-bonds are shown in purple color. (**a**) D18-c-KIT, (**b**) D23-c-KIT, (**c**) D25-c-KIT, (**d**) D28-c-KIT, (**e**) D32-c-KIT, (**f**) D39-c-KIT, (**g**) D44-c-KIT, (**h**) D45-c-KIT, **(i)** D18-PDGFRα, (**j**) D23-PDGFRα, (**k**) D25-PDGFRα, (**l**) D28-PDGFRα, (**m**) D32-PDGFRα, (**n**) D39-PDGFRα, (**o**) D44-PDGFRα, (**p**) D45-PDGFRα.

MD simulation of the eight designed compounds with the receptors was performed for 70 ns. The binding interactions of the designed compounds with the receptors are shown in Figure 3. The RMSD plots of the designed compounds during the simulation are shown in Figure S4 (Supplementary Material). The results showed that the designed compounds were able to form stable interactions with both c-KIT and PDGFRα throughout the simulation. In the designed compounds, the pyrazolopyridine moiety was anchored near the hinge region of c-KIT and PDGFRα through H-bond interactions with Cys673 and Cys677. The eight designed compounds also showed an H-bond interaction with DFG motif residues Asp810/Asp836 (c-KIT/PDGFRα). Except for compounds D23, D25, and D44 in PDGFRα, the designed compounds also formed weak H-bond interactions with the Lys623/Lyss627 (c-KIT/PDGFRα) and Glu640/Glu644 (c-KIT/PDGFRα).

The BE values of the designed compounds with both c-KIT and PDGFRα are given in Table 6. The evaluation of the BE values showed that the designed compounds possessed higher predicted binding affinity than imatinib and compound **14** for both receptors. Among the eight designed compounds, **D39** showed the highest binding affinity against both the receptors. In c-KIT, **D39** formed H-bond interactions with Cys673, Glu640, and Asp810. In PDGFRα, **D39** formed H-bond interactions with Cys677, Asp836, and Lys627. In addition, the benzyl and butyl substituents at the R1 and R2 positions of the methylbenzene extended into the hydrophobic pocket. This allowed the formation of hydrophobic interactions with residues Leu647, Val643, His790, Cys809, and Ile808 in c-KIT and Ile657, Met648, Val815, Leu809, and Ile834 in PDGFRα. The benzyl and butyl substituents were unique in **D39**, which suggested that having hydrophobic substituents in the R1 and R2 positions may increase the binding affinity toward both c-KIT and PDGFRα. The hydrophobic interactions of **D39** with the receptors are given in Figure S2 (Supplementary Materials).

The synthetic accessibility of the designed compounds was evaluated with SwissADMET (http://www.swissadme.ch/), and the results are given in Table S3 (Supplementary Material). The synthetic accessibilities of the compounds were scored within the range of 1 to 10 where a synthetic accessibility score of 1 indicates easy synthesis and a score of 10 indicates difficult synthesis. The designed compounds showed a reasonable synthetic accessibility score of less than 5. The absorption (A), distribution (D), metabolism (M), excretion (E), and toxicity (T) properties of the designed compounds were also evaluated using the pkCSM online server (http://biosig.unimelb.edu.au/pkcsm/) and the results are shown in Table S3 (Supplementary Materials) [40]. The steady-state volume of distribution (VDss) of a compound represents the degree to which the compound will likely get distributed in the body rather than the plasma. A VDss score is considered low if it is below −0.15 log L/kg. The designed compounds showed low to moderate VDss scores, suggesting a reasonable distribution rate. The designed compounds also showed a positive outcome for the cytochrome P450 substrate test, suggesting that the compounds are likely to be metabolized by cytochrome P450. Except for compounds **D32** and **D39**, the designed compounds showed a total clearance rate of at least 0.8. The compounds **D32** and **D39** showed a clearance rate of 0.6 and 0.5, respectively. The toxicity prediction showed that except for compound **D39**, the designed compounds tested negative for mutagenic potential.

## 3. Discussion

Molecular docking and molecular dynamics simulation of compound **14** showed H-bond interactions with Cys673, Glu640, and Asp810 in c-KIT and Cys677, Glu644, and Asp836 in PDGFRα. For comparative study, MD simulations of compound **31**, which showed low activity values for c-KIT (pIC_50_ = 6.2) and PDGFRα (pIC_50_ = 5.9), was performed. In c-KIT, compound **31** forms H-bond interactions with Cys673, Glu640, and Asp810, which were also observed in compound **14-c-KIT** interaction. However, compound **31** did not have the extended methylbenzene and morpholine moiety present in compound **14** and lost the hydrophobic interactions with Leu644, Ile768, Leu783, Leu647, Val643, and Ile808, which were observed in compound **14-c-KIT** interaction. The hydrophobic and H-bond interactions of compound **31** with c-KIT are shown in Figure S3 and Figure S5 (Supplementary Material) respectively. Similarly, compound **31** formed H-bond interactions with PDGFRα residues Cys677, Lys627, and Asp836, which were observed in the compound **31–PDGFRα** interaction. However, compound **31** did not form hydrophobic interactions at the catalytic loop and the αC-Helix due to the absence of the extended methylbenzene moiety. The loss of the hydrophobic interactions at the catalytic loop and the αC-Helix could be the reason why compound **31** showed lower activity value against both c-KIT and PDGFRα. 

Contour map analysis suggested that positive, bulky, and hydrophobic substituents were favored near the meta position of the methylbenzene of compound **14** and could increase activity for c-KIT and PDGFRα. The presence of bulky hydrophobic substituents at the meta position may lead to the formation of crucial hydrophobic interaction with residues from the αC-helix and the catalytic loop (Figure S5, Supplementary Material). The result is also supported by the BE evaluation, which showed that hydrophobic residues Leu644, Val643, and Leu647 from the αC-helix, and Leu783 and Cys788 from the catalytic loop made key contributions to the total BE in c-KIT (Table 3). Further analysis also showed that hydrophobic residues Val603, Leu644, Val654, Cys809, and Phe811 contributed more than −5 kJ/mol to the total BE in compound **14-c-KIT**. In contrast, the hydrophobic residues Val607, Met648, Val658, Leu825, and Cys835 contributed more than -5 kJ/mol to the total BE in compound **14-PDGFRα**. These results suggested that hydrophobic interactions were dominant in the binding of compound **14** with both the receptors. The eight designed compounds showed a higher binding affinity with both c-KIT and PDGFRα compared to compound **14** and imatinib. The higher binding affinity could be attributed to the hydrophobic substituents in the designed compounds, which were able to form interaction with hydrophobic residues from the catalytic loop and the αC-Helix (Figure S5a, Supplementary Material).

**Figure 4:**
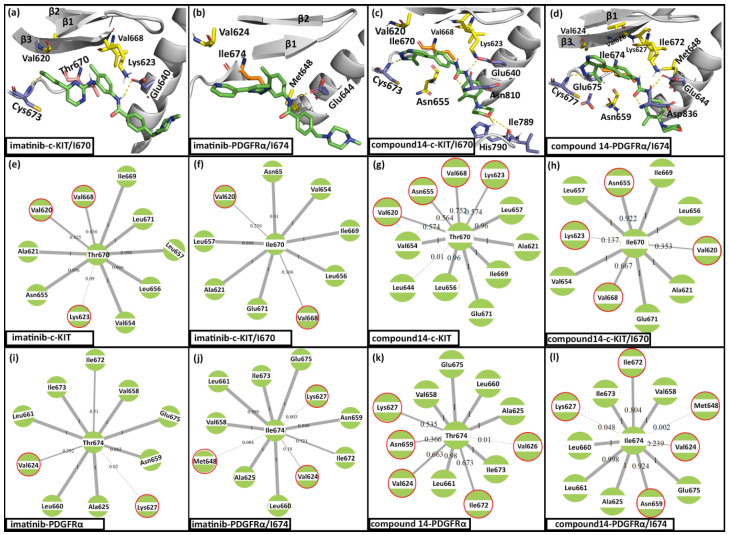
H-bond interactions and residue contact map from the MD simulation of imatinib and compound **14**. Proteins are shown in grey color cartoon representation and inhibitors are shown green color stick representation. H-bonds are shown in yellow dotted lines. Residues forming H-bond interactions are shown in purple color stick representation. Substituted residues and the residues forming interactions are shown in salmon color and yellow color stick representations respectively. H-bond interactions observed in (**a**) imatinib–c-KIT/I670, (**b**) imatinib–PDGFRα/I674, (**c**) compound **14–c-KIT/I670**, and (**d**) compound **14–PDGFRα/I674**. Residue contact map for (**e**) Thr670 in imatinib–c-KIT, (**f**) Ile670 in imatinib–c-KIT/I670, (**g**) Thr670 in compound **14–c-KIT**, (**h**) Ile670 in compound **14–c-KIT/I670**, (**i**) Thr674 in imatinib–PDGFRα, (**j**) Ile674 in imatinib–PDGFRα/I674, (**k**) Thr674 in compound **14–PDGFRα**, and (**l**) Ile674 in compound **14–PDGFRα/I674**. Weight on the edge between two residues represented how often the interaction existed between the residues during the simulation. Residues that showed a significant increase or decrease in interactions with the residue of interest are highlighted in red circles.

Earlier studies have shown that imatinib resistance is achieved via T670I substitution in c-KIT and T674I substitution in PDGFRα [19,39]. However, the molecular mechanisms underlying the drug resistance remained unclear. We have performed MD simulations of the imatinib with c-KIT/I670 and PDGFRα/I674 mutants to study the effect of the T670I/T674I substitutions on the binding interactions. The results showed that imatinib formed an H-bond interaction with Cys673 and Glu640 in c-KIT/I670. In the imatinib–PDGFRα/I674 complex, imatinib formed only one H-bond interaction with Glu644 and the pyridine of imatinib moved out of the hinge region. This outward movement could be attributed to the loss of H-bond interaction with Cys677 as a result of the T674I substitution. The binding interaction of imatinib and compound **14** with c-KIT/I670 and PDGFRα/I674 are shown in Figure 4. In c-KIT/I670, compound **14** formed H-bond interactions with Cys673, Glu640, Asn810, Ile789, and His790. In contrast, compound **14** formed H-bond interactions with Cys677, Glu675, Glu644, and Asp836 in PDGFRα/I674. Following the T670I substitution, the total BE of imatinib was reduced from −105 kJ/mol (wild-type) to −72 kJ/mol (c-KIT/I670). Similarly, the total BE value of imatinib reduced from −104 kJ/mol (wild-type) to −67 kJ/mol (PDGFRα/I674) after T674I substitution. On the other hand, compound **14** showed total BE values of −131 kJ/mol with c-KIT/I670 and −122 kJ/mol with PDGFR/I674. These results suggested that the T670I/T674I substitutions disrupted the interaction of imatinib with c-KIT/I670 and PDGFR/I674, which consequently reduced the binding affinity against the receptors. In contrast, compound **14** was able to retain the interactions with c-KIT/I670 and PDGFRα/I674, resulting in high binding affinity against both receptors. In contrast to the H-bond interaction between the amide linker of imatinib and T670/T674 in c-KIT/PDGFRα, compound **14** formed hydrophobic interactions with T670/T674 (Figure S2, Supplementary Material). Hence, the substitution of the hydrophilic threonine residue with the hydrophobic isoleucine residue could have led to the loss of H-bond interaction with imatinib while it increased the binding affinity for compound **14** through hydrophobic interactions. 

Residue contact map analysis was calculated for the Ile670 (c-KIT/I670) and Ile674 (PDGFRα/I674) to study the effect of the T670I/T674I substitutions on the residue interactions at the binding sites. The residue contact map shows how often a residue of interest interacted with its surrounding residues throughout the simulation [41]. The I670/I674 contact maps were generated from the MD trajectories of imatinib and compound **14** with c-KIT, c-KIT/I670, PDGFRα, and PDGFRα/I674. The contact maps are shown in Figure 4. Comparison of the Thr670 (imatinib–c-KIT) and the Ile670 (imatinib–c-KIT/I670) contact maps showed that the T670I substitution led to the loss of interactions with the Val620 and Val668 (Figure 4e,f). Similarly, the T674I substitution also led to the loss of interactions with Val672 and Lys627 in imatinib–PDGFRα/I674 (Figure 4i,j). The comparison of the Thr670 (compound **14–c-KIT**) and Ile670 (compound **14–c-KIT/I670**) contact maps showed that the substitution led to the loss of interactions with Val668, Val620, and Lys623. However, the substitution has also led to more interactions with Asn655. Comparison of the contact maps for Thr674 and Ile674 in compound **14–PDGFRα** and compound **14–PDGFRα/ILE674** showed that that the substitutions led to the loss of interaction with Val624, Val626, and Lys627. However, T674I substitution also led to more interaction with Asn659, Ile672, and Met648. 

These results suggested that the T670I substitution in c-KIT induced conformation changes at the binding site which led to reduced interactions with Val620, Val668, and Lys623 while it increased the interactions with Asn655. Similarly, T674I substitution in PDGFRα led to the loss of interaction with Val624 and Lys627. The loss of interaction with Val620/Val624 and Lys623/Lys627 in c-KIT/PDGFRα upon T670I/T674I substitution was characterized by the movement of the I670/I674 at the DFG motif away from the β3 and moving closer to the αC-helix. These results complemented earlier claims that the T670I/T674I substitutions modified the binding pocket of c-KIT/PDGFRα [19,42]. 

## 4. Methodology

### 4.1. Data Preparation

The dataset of 48 pyrazolopyridine derivatives and their inhibitory values against c-KIT and PDGFRα were collected for computational study [39]. The half-maximal inhibitory concentration (IC_50_) values of the compounds were converted to its log (pIC_50_) values. The compounds were sketched and minimized in Sybyl X 2.1 (Tripos, St. Louis, MO, USA). Compound **14**, which showed the highest activity for both c-KIT (pIC_50_ = 8.6) and PDGFRα (pIC_50_ = 8.1), was selected as a representative compound for the dataset.

The X-ray crystal structure of the imatinib–c-KIT complex (PDB ID 1T46) and imatinib–PDGFRα complex (PDB ID 6JOL) were collected from the protein databank (https://www.rcsb.org) [5]. The water molecules, ions, and other small molecules were removed from the protein file. The missing residues of the proteins were modeled using the SWISS-MODEL (https://swissmodel.expasy.org/) [43].

### 4.2. Molecular Docking

The binding interactions of compound **14** with the c-KIT and PDGFRα were studied using Autodock 4.2 (Scripps Research, CA, USA) [44]. The receptor was prepared by removing the heteroatoms. This was followed by the addition of hydrogen atoms and the application of partial charges to all the atoms. The inhibitor was prepared by assigning partial charges and the number of rotatable bonds. A grid box of 70 × 70 × 70 was developed to define the search space inside the receptor using the Autogrid program. The Lamarckian genetic algorithm was selected to perform the docking of the ligand. To validate the docking protocol, the crystal ligand (imatinib) was minimized outside the receptor and docked into the receptor. The docking results showed that the docked pose closely overlapped with the crystal ligand in both c-KIT and PDGFRα, as shown in Figure S1 (supplementary materials).

The result of the docking was analyzed using the autodock tools (Scripps Research, CA, USA). This docking protocol was used for all protein–ligand interactions in this study.

### 4.3. Molecular Dynamics Simulation

The protein–ligand dynamics simulation was performed with the Gromacs 2020 [45,46,47]. The protein–ligand complex from the molecular docking study was used as the initial structure for the MD simulation. The protein parameter files were generated with the CHARMM36 all-atom force field (2019) [48]. The ligand topology and parameter files were prepared using the CHARMM General Force Field (CGenFF) (Massachusetts, USA) [49]. Initially, the system was set up containing the protein–ligand complex inside a dodecahedron box and solvated with TIP3 waters. The charge of the system was neutralized by adding Na^+^ and Cl^−^ counterions. A steepest descent energy minimization step was performed to remove steric clashes and inappropriate geometries. This was followed by 100 ps isothermal–isochoric ensemble (NVT ensemble) equilibration and 100 ps isothermal–isobaric ensemble (or NPT ensemble) equilibration of the system to stabilize the water around the protein and ligand. The protein was kept restrained during the equilibrations. Temperature and pressure coupling was performed using Berendsen thermostat (Groningen, Netherlands) and Parrinello–Rahman barostat (Argonne, IL, USA), respectively. Long-range electrostatics were treated using the Particle-mesh Ewald method. The thermodynamic properties of the system were collected every 1 ps. The unrestrained MD production run was performed for 100 ns at the temperature and pressure of 300 K and 1 bar, respectively. 

### 4.4. Evaluation of Binding Energy

The binding energy between the protein and the ligand was calculated using the g_mmpbsa package (New Delhi, India) [50]. Molecular mechanics energies combined with the Poisson–Boltzmann and surface area continuum solvation (MM/PBSA) methods have been successfully used to predict the relative binding energy values of congeneric compounds. In g_mmpbsa, the MM potential energy term is calculated based on the molecular mechanics force-field parameters, as given in the Equation (1).

(1) $$E_{MM}=E_{bonded}+E_{nonbonded}=E_{bonded}+\left(E_{vdw}+E_{elec}\right)$$

where *E*_bonded_ is a bonded energy term consisting of bond, angle, dihedral, and improper interactions. The non-bonded energy term is made up of electrostatics and van der Waals energy terms and was calculated based on Coulomb and Lennard–Jones potential functions, respectively. The free energy of solvation was calculated based on an implicit solvent model where the electrostatic (*G_polar_*) and non-electrostatic (*G_nonpolar_*) energy terms were calculated as given Equation (2).

(2) $$G_{MM}=G_{polar}+G_{nonpolar}$$

The electrostatic term and non-electrostatic terms were calculated based on the Poisson–Boltzmann equation and solvent-accessible surface area (SASA) model, respectively.

Before calculating the binding energy, the trajectory was processed to correct the periodicity and center the protein within the unit cell. Water molecules and ions were removed from the trajectory file. Finally, the binding energy was calculated from the converged region of the MD trajectory at an interval of 0.5 ns (100 frames).

### 4.5. 3D-QSAR

To perform the 3D-QSAR studies, we have selected a binding conformation of compound **14** from the MD simulation trajectories. A conformation that corresponded to the highest number of H-bond interactions with the receptor was selected. The structure of the compound was relaxed by quick minimization in Sybyl X 2.1. Using compound **14** as a template, the other compounds were sketched and minimized. The alignment of the compounds was performed based on the common substructure of the compounds [37,38]. The aligned compounds were randomly divided into a training set and a test set.

The training set compounds were used to develop various comparative molecular field analysis (CoMFA) and comparative molecular similarity indices analysis (CoMSIA) models [51,52]. In CoMFA, the electrostatic and van der Waal energy terms were calculated for each compound. In CoMSIA, the hydrogen bond donor, hydrogen bond acceptor, hydrophobic, steric, and electrostatic energy terms were calculated for each compound [53]. The energy terms were calculated by probing the 3D grid around the compound by using an sp^3^ hybridized carbon atom (charge +1). The partial least square (PLS) method was used to establish the relationship between the dependent and the independent variables. Leave-One-Out crossvalidated analysis was performed to determine the predictive ability of the 3D-QSAR models and to determine the optimal number of components (ONC). Based on the crossvalidated *q^2^* and the ONC, the non-crossvalidated predictive *r^2^* value was calculated. Based on the statistical results, a model with high *q^2^* and *r^2^* values was selected for further analysis.

Bootstrapping (BS) was performed to estimate the confidence intervals of the parameters predicted by the 3D-QSAR models. A bootstrap sampling size of 100 was used during the validation. In addition, the predictive ability of the derived models against an external test set was also evaluated using the equation given Equation (3).

(3) rpred2=(SD−PRESS)SD

where *SD* represented the standard deviation between the activity value (pIC50) of the test set compounds and the mean activity value of the training set compounds. *PRESS* represented the sum of the square deviation between the predicted and the actual activity value of each compound in the test set.

## 5. Conclusions

We performed a modelling study of pyrazolopyridine derivatives that showed inhibitory activity for both c-KIT and PDGFRα. A 3D-QSAR study was performed to understand the structural properties important for the dual inhibition of c-KIT and PDGFRα. Contour maps analysis showed that positive and bulky substituents are favorable near the meta and para position of compound **14** and may lead to an increase in activity against c-KIT and PDGFRα. In contrast, bulky substituents near the ortho position of reference compound **14** were not favored and could lead to steric clashes with binding site residues in both receptors. A comparative study of compound **14** and compound **31** (low activity value for c-KIT/PDGFRα) showed that compound **14** was able to form additional interactions at the hydrophobic pocket formed by residues from the catalytic loop and the αC-helix. These interactions were not observed in the interactions of compound **31** with c-KIT/PDGFRα. The results suggested that possessing substituents that extended into the hydrophobic pocket could be crucial to increase the activity against c-KIT and PDGFRα. Based on the predicted activity values from the 3D-QSAR models, eight compounds were selected as potential c-KIT/PDGFRα inhibitors. The eight designed compounds showed higher BE values against c-KIT/PDGFRα than imatinib. Residue contact map analysis indicated that the T670I/T674I substitution in c-KIT/ PDGFRα led to conformational changes at the binding sites. BE calculation showed that following the T670I/T674I substitution, the activity values of imatinib against c-KIT/I670 and PDGFRα /I674 reduced to −72 kJ/mol and −67 kJ/mol, respectively. In contrast, the activity values of compound **14** against c-KIT and PDGFRα were −131 kJ/mol and −126 kJ/mol, suggesting that compound **14** was able to retain the activity values against both receptors after the T670I/T674I substitutions. The high activity values of compound **14** against both wild-type and mutant c-KIT/PDGFRα showed the potential of the pyrazolopyridine derivatives as c-KIT/PDGFRα inhibitors for the treatment of imatinib-resistant GIST. The outcome of this study could provide valuable insight into designing more potent dual c-KIT and PDGFRα inhibitors.

## Figures and Tables

**Table 1:** Structure of the pyrazolopyridine derivatives and their pIC_50_ values for stem cell factor receptor (c-KIT) and platelet-derived growth factor receptor alpha (PDGFRα).

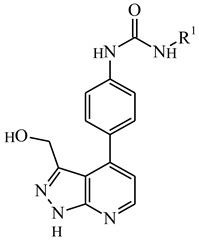				
Structure A				
Compounds	Structures	R1	c-KIT (pIC_50_)	PDGFRα (pIC_50_)
**1**	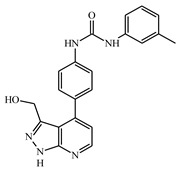		8.62	7.06
**2**	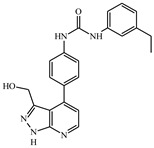		8.43	7.49
**3**	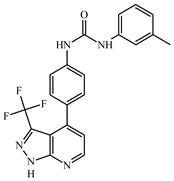		>4.3	>4.3
**4**	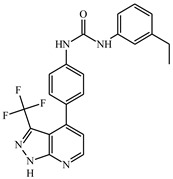		5.2	>4.3
**5**	A	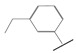	8.41	7.66
**6**	A	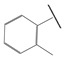	4.96	6.52
**7**	A	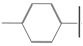	5.74	7.19
**8**	A	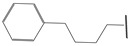	5.32	6.58
**9**	A	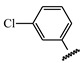	5.62	6.74
**10**	A	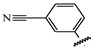	6.70	4.67
**11**	A	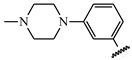	7.72	6.67
**12**	A	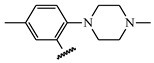	5.71	5.75
**13**	A	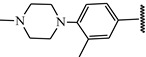	8.14	6.90
**14**	A	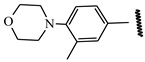	8.62	8.14
**15**	A	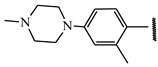	5.65	5.96
**16**	A	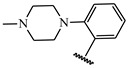	5.34	6.03
**17**	A	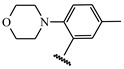	6.40	4.72
**18**	A	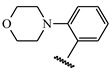	5.19	5.39
**19**	A	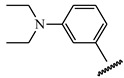	7.92	6.87
**20**	A	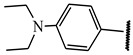	7.92	6.75
**21**	A	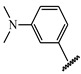	8.59	7.08
**22**	A	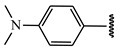	8.03	6.51
**23**	A	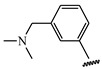	4.66	5.34
**24**	A	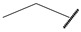	4.4	>4.3
**25**	A	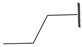	4.7	>4.3
**26**	A	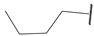	4.69	4.36
**27**	A	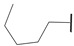	8.46	6.79
**28**	A	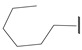	8.85	7.57
**29**	A	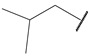	6.49	5.95
**30**	A	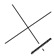	5.85	5.83
**31**	A	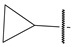	6.26	5.95
**32**	A	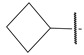	6.50	6.14
**33**	A	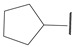	6.84	6.24
**34**	A	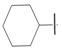	>4.3	>4.3
**35**	A	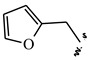	7.29	6.41
**36**	A	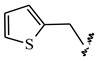	7.59	6.80
**37**	A	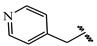	7.11	6.33
**38**	A	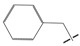	>4.3	>4.3
**39**	A	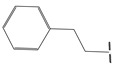	8.43	7.09
**40**	A	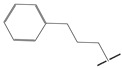	8.77	7.62
**41**	A	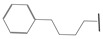	8.21	6.87
**42**	A	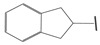	8.06	7.08
**43**	A	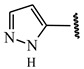	6.92	6.39
**44**	A	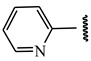	7.70	6.40
**45**	A	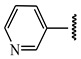	7.85	6.50
**46**	A	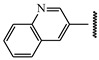	8.72	7.66
**47**	A	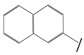	7.70	6.91
**48**	A	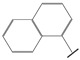	8.44	7.01

**Table 2:** The energy contribution of the various energetic terms (Van der Waals energy, electrostatic energy, polar solvation energy, and non-polar solvation energy/solvent-accessible surface area (SASA)) to the total binding energy during the binding of imatinib and compound **14** with c-KIT and PDGFRα.

Complexes	Van der Waals (kJ/Mol)	Electrostatics (kJ/Mol)	Polar Solvation (kJ/Mol)	SASA (kJ/Mol)	Total Binding Energy (kJ/Mol)
**Imatinib–c-KIT**	−260	−74	257	−28	−105
**Imatinib–PDGFRα**	−244	−58	225	−27	−104
**Compound 14–c-KIT**	−257	−57	219	−25	−120
**Compound 14–PDGFRα**	−251	−55	213	−25	−118
**Compound 31–c-KIT**	−183	−48	185	−19	−65
**Compound 31–PDGFRα**	−180	−50	163	−18	−85
**Imatinib–c-KIT/I670**	−227	−48	228	−26	−73
**Imatinib–PDGFRα/I674**	−248	−18	227	−28	−67
**Compound 14–c-KIT/I670**	−250	−61	205	−25	−131
**Compound 14–PDGFRα/I674**	−265	−39	203	−25	−126

**Table 3:** Residues that showed a high contribution to the total binding energy during the MD simulations of **Compound 14**–**c-KIT**, **imatinib–c-KIT**, **Compound 14**–**PDGFRα** and imatinib–PDGFRα. The energy values of the residues are given in kJ/mol.

c-KIT Residues	Compound 14–c-KIT (kJ/mol)	Imatinib–c-KIT (kJ/mol)	PDGFRα Residues	Compound 14–PDGFRα (kJ/mol)	Imatinib–PDGFRα (kJ/mol)
Asp572	−0.9	−0.7	Glu587	−0.66	−0.42
Leu595	−2.6	−3.4	Leu599	−2.48	−2.87
Val603	−5.4	−3.2	Gly600	−0.91	−0.82
Ala621	−2.7	−3.1	Val607	−5.23	−5.10
Val620	−0.7	−0.8	Val608	−1.10	−0.69
Val622	−1.0	−1.7	Glu609	−1.31	−2.33
Glu635	−0.8	−0.7	Val624	−0.76	−1.10
Val643	−3.1	−3.6	Ala625	−2.20	−1.87
Leu644	−6.7	−6.6	Val626	−1.15	−1.84
Leu647	−2.1	−0.5	Glu637	−0.83	−0.96
Ile653	−3.0	−0.9	Ile647	−4.19	−4.35
Val654	−5.2	−7.6	Met648	−8.93	−7.98
Tyr672	−3.9	−5.6	Leu651	−2.11	−0.96
Cys673	−2.1	−2.5	Ile657	−2.52	−0.76
Gly676	−0.8	−0.4	Val658	−5.46	−5.28
Leu783	−2.9	−0.6	Ile672	−0.83	−2.95
Cys788	−1.3	−1.8	Tyr676	−3.39	−4.99
His790	−3.6	−1.9	Cys677	−2.48	−1.82
Asp792	−1.0	−0.2	Gly680	−0.92	−0.76
Leu799	−4.9	−4.3	Leu809	−2.58	−1.14
Lys807	−1.1	2.2	Cys814	−2.27	−1.81
Ile808	−1.0	0.4	Leu825	−5.59	−5.59
Cys809	−6.4	−6.2	Ile834	−1.21	−0.40
Asp810	−2.1	9.3	Cys835	−6.27	−5.11
Phe811	−6.8	−4.0	Asp836	−5.11	1.89
Asp851	−1.5	−0.5	Phe837	−4.87	−7.16

**Table 4:** Statistical results of the comparative molecular field analysis (CoMFA) and comparative molecular similarity indices analysis (CoMSIA) models for c-KIT and PDGFRα.

Parameters	CoMFA (c-KIT)	CoMSIA (c-KIT)	CoMFA (PDGFRα)	CoMSIA (PDGFRα)
*q* ^2^	0.63	0.6	0.61	0.62
ONC	6	5	6	3
*r* ^2^	0.98	0.9	0.98	0.81
SEE	0.2	0.46	0.12	0.39
F value	204	43	232	46
BS *r*^2^	0.98	0.94	0.98	0.97
BS SD	0.15	0.32	0.1	0.14
*r* ^2^ *_pred_*	0.59	0.58	0.56	0.59
Influence of different fields (%)				
S	59	50	67	42
E	41	-	33	-
H	-	50	-	58

*q*^2^: cross-validated correlation coefficient; ONC: optimal number of components; *r*^2^: non-cross-validated correlation coefficient; SEE: standard error of estimation; F value: F-test value; *r*^2^; BS-*r*^2^: bootstrapping *r*^2^ mean; BS-SD: bootstrapping standard deviation; *r*^2^*_pred_*: predictive correlation coefficient; S: steric; E: electrostatic; H: hydrophobic; -not applicable.

**Table 5:** The chemical structures and the predicted pIC_50_ values of the newly designed compounds for c-KIT and PDGFRα.

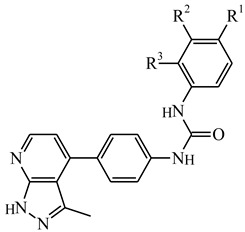					
Compounds	R1	R2	R3	Predicted Activity (pIC_50_)	
				c-KIT	PDGFRα
Compound **D18**	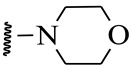	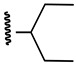	H	10.4	8.3
Compound **D23**	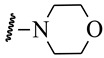	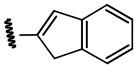	CH3	10.1	8.2
Compound **D25**	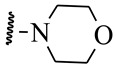	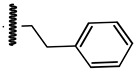	CH3	10.5	8.1
Compound **D28**	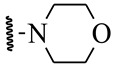	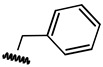	CH3	9.6	8.4
Compound **D32**	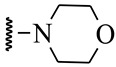	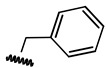	CH3	9.1	8.3
Compound **D39**	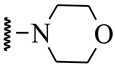	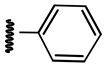	H	10.3	8.1
Compound **D44**	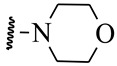	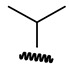	H	10.2	8.3
Compound **D45**	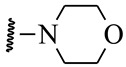	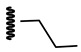	H	9.3	8.1

**Table 6:** The energy contributions of the various energetic terms (Van der Waals energy, electrostatic energy, polar solvation energy, and non-polar solvation energy/SASA) to the total binding energies are shown for the designed compounds.

Complexes (Designed Compounds–Receptor)	Van der Waals (kJ/Mol)	Electrostatics (kJ/Mol)	Polar Solvation (kJ/Mol)	SASA (kJ/Mol)	Total Binding Energy (kJ/Mol)
**D18–c-KIT**	−272	−57	218	−28	−139
**D18–PDGFRα**	−282	−57	224	−27	−142
**D23–c-KIT**	−287	−48	242	−29	−122
**D23–PDGFRα**	−286	−36	212	−28	−138
**D28–c-KIT**	−278	−62	242	−27	−126
**D28–PDGFRα**	−289	−38	227	−29	−129
**D32–c-KIT**	−271	−53	229	−27	−122
**D32–PDGFRα**	−283	−44	224	−28	−130
**D39–c-KIT**	−268	−65	212	−26	−148
**D39–PDGFRα**	−274	−48	200	−26	−150
**D44–c-KIT**	−262	−56	216	−25	−129
**D44–PDGFRα**	−261	−42	209	−26	−120
**D45–c-KIT**	−262	−76	244	−26	−121
**D45–PDGFRα**	−274	−58	215	−26	−143

## Supplementary Materials

Supplementary materials can be found at https://www.mdpi.com/1422-0067/21/21/8232/s1. Figure S1: Showing the H-bond interactions of imatinib and compound **14** with c-KIT and PDGFRα from the molecular docking studies. Figure S2: Showing the hydrophobic interactions of the inhibitors with c-KIT and PDGFRα. Figure S3: Scatter plot for the CoMFA and CoMSIA models. Figure S4: The ligand RMSD plots from the MD simulations. Figure S5: Hydrophobic surface of c-KIT and the binding interactions of compound **31** with the receptors. Table S1: The experimental/actual and predicted pIC_50_ values with their residuals for the CoMFA and CoMSIA for c-KIT. Table S2: The experimental/actual and predicted pIC_50_ values with their residuals for the CoMFA and CoMSIA for PDGFRα. Table S3: The predicted ADMET values and synthetic accessibility values for the 8 designed compounds.

## Abbreviations

S	Steric
E	Electrostatic
H	Hydrophobic
ADMET	Absorption, distribution, metabolism, excretion and toxicity
BE	Binding energy
BS SD	Bootstrap standard deviation
CADD	Computer-aided drug discovery
c-KIT	Stem cell factor receptor
CoMFA	Comparative molecular field analysis
CoMSIA	Comparative molecular similarity indices analysis
FDA	Food and Drug Administration
FGFR	Fibroblast growth factor receptor
FLT3	Fms like tyrosine kinase 3
GISTs	Gastrointestinal stromal tumors
ICC	Interstitial cells of Cajal
MD	Molecular dynamics
MM/PBSA	Molecular mechanics energies combined with the Poisson–Boltzmann and surface area continuum solvation
ONC	Optimal number of components
PDB	Protein data bank
PDGFRa	Platelet derived growth factor receptor alpha
RAF1	Rapidly accelerated fibrosarcoma 1
RET	Rearranged during transfection
RMSD	Root mean square deviation
SASA	Solvent accessible surface area
SEE	Standard error of estimation
VEGFR	Vascular endothelial growth factor receptor
3D-QSAR	Three-dimensional quantitative structure–activity relationship
