# Supplementary material for: Molecular Modeling Study of c-KIT/PDGFRα Dual Inhibitors for the Treatment of Gastrointestinal Stromal Tumors
Source: Int J Mol Sci. 2020 Nov 3;21(21):8232. doi: 10.3390/ijms21218232 (PMC7662224; doi:10.3390/ijms21218232)

Supplementary Materials

Molecular Modelling Study of c-KIT/PDGFRα Dual Inhibitors for the Treatment of Gastrointestinal Stromal Tumors

Seketoulie Keretsu[1], Suparna Ghosh[1], Seung Joo Cho[1,2,*]

[1]*Department of Biomedical Sciences, College of Medicine, Chosun University, Gwangju 501-759, Republic of Korea; keretsu@chosun.kr(S.K); s.ghosh@chosun.kr (S.G);* chosj@chosun.ac.kr (S.J.C)

[2] *Department of Cellular Molecular Medicine, College of Medicine, Chosun University, Gwangju 501-759, Republic of Korea*

Table **S1**. The experimental/actual and predicted pIC_{50} values with their residuals for the CoMFA and CoMSIA for c-KIT.

Compound	Experimental pIC_{50}	c-KIT CoMFA		c-KIT CoMSIA	
		Predicted pIC_{50}	Residual	Predicted pIC_{50}	Residual
**1**	8.62	8.27	-0.35	8.27	0.35
**2**	8.43	8.61	0.17	9.10	-0.67
**5**	8.41	8.58	0.17	8.13	0.28
**6**[*]	4.96	5.80	0.84	5.96	-0.99
**7**	5.74	5.91	0.17	6.73	-0.99
**8**	5.32	5.26	-0.06	5.86	-0.54
**9**	5.62	5.51	-0.11	5.51	0.11
**10**[*]	6.70	7.21	-0.52	7.33	-0.63
**11**	7.72	7.62	-0.10	7.57	0.15
**12**	5.71	5.87	0.16	5.88	-0.17
**13**	8.14	8.16	0.02	8.18	-0.04
**14**	8.62	8.68	0.06	8.27	0.35
**15**[*]	5.65	6.55	-0.90	6.60	-0.95
**16**	5.34	5.17	-0.17	5.16	0.18
**17**	6.40	6.12	-0.28	6.07	0.33
**18**	5.19	5.50	0.31	5.35	-0.16
**19**	7.92	7.97	0.05	8.26	-0.34
**20**[*]	7.92	7.62	0.30	8.90	-0.98
**21**	8.59	8.71	0.13	8.41	0.17
**22**	8.03	7.86	-0.17	7.78	0.25
**23**[*]	4.66	5.58	-0.93	5.60	-0.94
**26**	4.69	4.73	0.04	5.31	-0.62
**27**	8.46	8.37	-0.09	8.51	-0.05
**28**[*]	8.85	8.09	0.77	8.44	0.42
**29**	6.49	6.41	-0.08	5.88	0.62
**30**	5.85	6.10	0.25	5.82	0.03
**31**	6.26	6.18	-0.08	5.93	0.32
**32**[*]	6.50	6.46	0.03	6.30	0.20

**33**	6.84	6.94	0.10	7.08	-0.24
**35***	7.29	8.10	-0.81	7.92	-0.62
**36**	7.59	7.48	-0.10	7.45	0.14
**37***	7.11	6.11	1.00	6.47	0.64
**39**	8.43	8.57	0.14	8.37	0.06
**40**	8.77	9.02	0.25	8.88	-0.11
**41**	8.21	8.32	0.11	8.71	-0.51
**42***	8.06	8.91	-0.85	8.68	-0.62
**43**	6.92	6.98	0.06	7.15	-0.23
**44**	7.70	7.75	0.05	7.75	-0.05
**45***	7.85	6.92	0.94	7.62	0.23
**46**	8.72	8.37	-0.35	8.45	0.28
**47***	7.70	8.42	-0.72	8.49	-0.79
**48**	8.44	8.14	-0.30	7.52	0.92

* Test set compounds

Table **S2**. The experimental/actual and predicted pIC_{50} values with their residuals for the CoMFA and CoMSIA for PDGFRα.

**Compound**	**Experimental pIC_{50}**	PDGFRα **CoMFA**		PDGFRα **CoMSIA**	
		**Predicted pIC_{50}**	**Residual**	**Predicted pIC_{50}**	**Residual**
**1**	7.06	7.14	-0.08	7.32	-0.26
**2**	7.49	7.49	0.01	7.45	0.04
**5**	7.66	7.54	0.12	7.23	0.43
**6***	6.52	6.11	0.40	6.78	-0.27
**7**	7.19	6.98	0.21	6.99	0.20
**8**	6.58	6.55	0.03	6.81	-0.22
**9**	6.74	6.87	-0.13	7.05	-0.31
**10***	4.67	3.85	0.82	5.56	-0.89
**11**	6.67	6.70	-0.03	6.57	0.09
**12**	5.75	5.57	0.18	5.25	0.50
**13**	6.90	7.00	-0.11	6.96	-0.06
**14**	8.14	7.98	0.16	7.96	0.18
**15***	5.96	6.34	-0.38	6.65	-0.69
**16**	6.03	6.25	-0.22	5.50	0.53
**17**	4.72	4.66	0.06	5.30	-0.57
**18**	5.39	5.35	0.04	5.56	-0.17
**19**	6.87	6.89	-0.03	7.05	-0.19
**20***	6.75	6.81	-0.06	7.04	-0.29
**21**	7.08	7.04	0.04	6.97	0.11
**22**	6.51	6.54	-0.03	6.86	-0.35
**23***	5.34	5.99	-0.66	6.17	-0.83
**26**	4.36	4.33	0.03	4.59	-0.23
**27**	6.79	6.86	-0.07	6.80	-0.01
**28***	7.57	6.94	0.63	6.90	0.67
**29**	5.95	5.96	-0.01	5.78	0.17
**30**	5.83	6.09	-0.25	5.90	-0.07
**31**	5.95	5.91	0.04	6.00	-0.06

**32***	6.14	5.88	0.26	5.80	0.34
**33**	6.24	6.20	0.04	5.73	0.51
**35***	6.41	6.88	-0.46	6.18	0.23
**36**	6.80	6.80	0.00	6.47	0.34
**37***	6.33	6.86	-0.53	6.27	0.05
**39**	7.09	7.08	0.01	7.45	-0.36
**40**	7.62	7.66	-0.04	7.33	0.29
**41**	6.87	7.05	-0.18	6.90	-0.03
**42***	7.08	7.54	-0.46	6.97	0.11
**43**	6.39	6.29	0.10	6.16	0.23
**44**	6.40	6.41	-0.01	6.43	-0.03
**45***	6.50	6.29	0.22	6.03	0.47
**46**	7.66	7.57	0.09	7.50	0.16
**47***	6.91	6.21	0.70	6.61	0.30
**48**	7.01	6.99	0.03	6.43	0.58

* Test set compounds

Table S3: The predicted ADMET values and synthetic accessibility values for the 8 designed compounds.

Compounds	Absorption	Distribution	Metabolism							Excretion	Toxicity	Synthetic Accessibility
	Intestinal absorption (human)	VDss (human)	CYP							Total Clearance	AMES toxicity	
			**2D6**	**3A4**	**1A2**	**2C19**	**2C9**	**2D6**	**3A4**			
			substrate		inhibitor							
	Numeric (% Absorbed)	Numeric (log L/kg)	Categorical (Yes/No)							Numeric (log ml/min/kg)	Categorical (Yes/No)	
**D18**	89	0.2	Yes	Yes	No	Yes	Yes	No	Yes	0.9	No	3.8
**D23**	98	-0.5	Yes	Yes	Yes	Yes	No	Yes	No	0.8	No	4.4
**D25**	87	-0.4	Yes	Yes	No	No	No	No	No	0.8	No	4.0
**D28**	92	-0.7	Yes	Yes	Yes	Yes	Yes	No	Yes	0.8	No	3.9
**D32**	93	-0.8	Yes	Yes	Yes	Yes	Yes	No	No	0.6	No	3.8
**D39**	88	-1.2	Yes	Yes	Yes	Yes	No	No	No	0.5	Yes	3.5
**D44**	87	0.1	Yes	Yes	Yes	Yes	Yes	No	No	0.8	No	3.5
**D45**	86	0.2	Yes	Yes	Yes	Yes	Yes	No	Yes	0.9	No	3.6

Figure S1. Showing the H-bond interactions of **imatinib** and **compound 14** with c-KIT and PDGFRα from the molecular docking studies. H-bond interactions were represented by yellow dotted lines and residues forming H-bonds were shown in purple color. **(a)** Binding interactions between imatinib (green) and c-KIT. The overlap between the docked pose (green) and the crystal ligand (salmon) is shown in inset **(b)** Binding interactions between imatinib (magenta) and PDGFRα. The overlap between the docked pose (magenta) and the crystal ligand (salmon) is shown in inset **(c)** Binding interactions between compound 14 (cyan) and c-KIT. **(d)** Binding interactions between compound 14 (yellow) and PDGFRα.

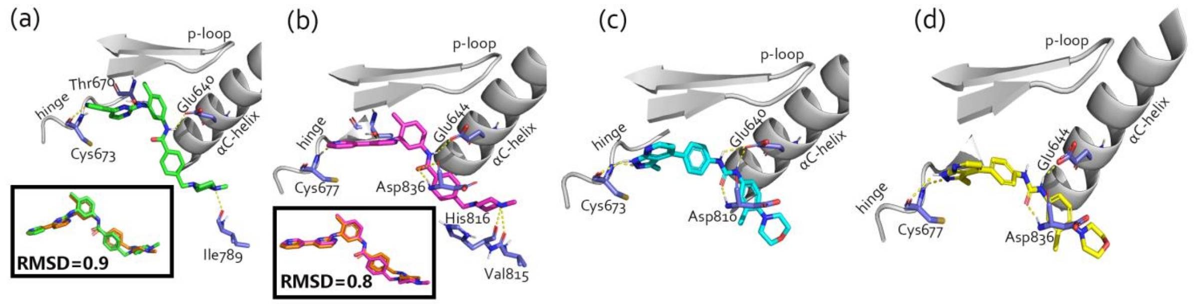

Figure S2. Showing the hydrophobic interactions of the inhibitors with c-KIT and PDGFRα. Hydrophobic interactions were represented in red dotted lines. Residues that showed Hydrophobic and H-bond interactions were given in green and black label respectively. **(a) Compound 14**-c-KIT **(b) Compound 31**-c-KIT **(c) D39**-c-KIT **(d) compound D39**-PDGFRα.

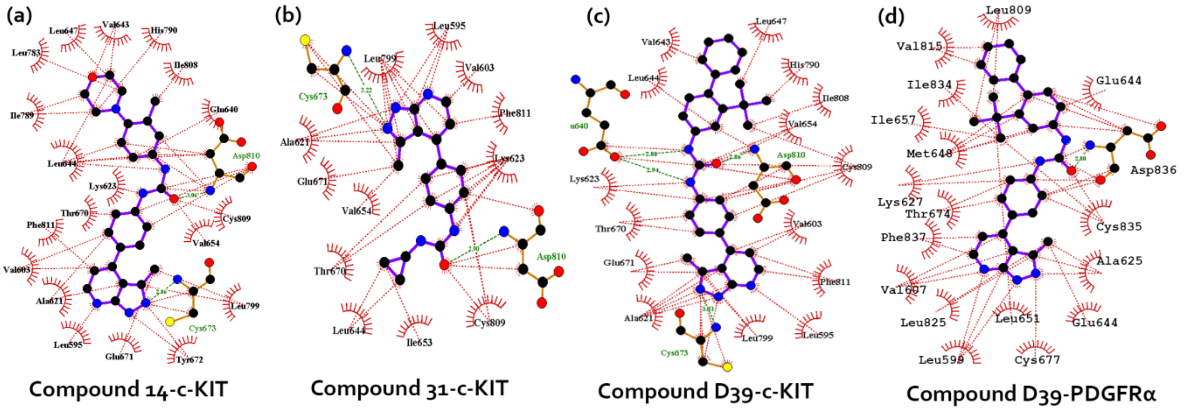

Figure S3. Scatter plot for the CoMFA and CoMSIA models. Scatter plot for **(a)** c-KIT CoMFA model **(b)** c-KIT CoMSIA model **(c)** PDGFRα CoMFA model **(d)** PDGFRα CoMSIA model. The values on the x-axis and y-axis represent the predicted pIC_{50} values and the experimental/actual pIC_{50} values respectively.

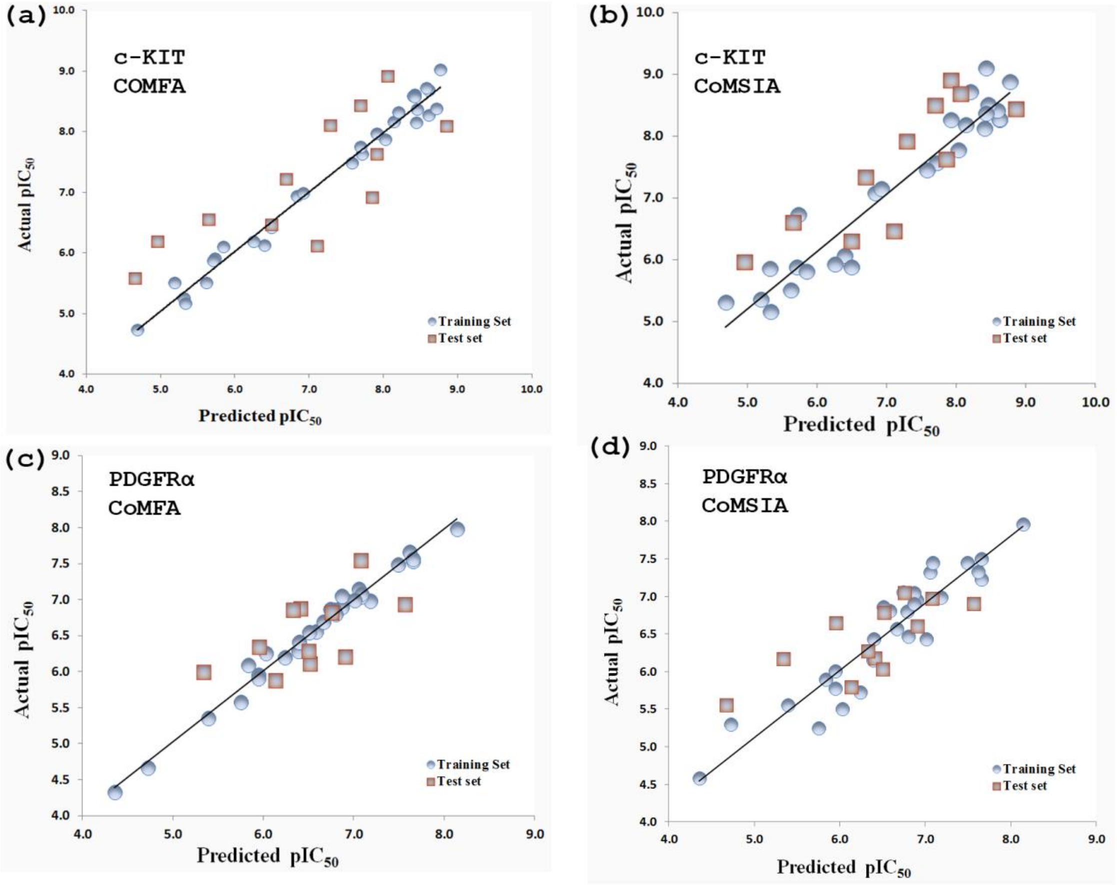

Figure S4. The ligand RMSD plots from the MD simulations. **(a)** Imatinib-c-KIT **(b)** Compound 14-c-KIT **(c)** Compound 31-c-KIT **(d)** D18-c-KIT **(e)** D23-c-KIT **(f)** D25-c-KIT **(g)** D28-c-KIT **(h)** D32-c-KIT **(i)** D39-c-KIT **(j)** D44-c-KIT **(k)** D45-c-KIT **(l)** Imatinib-c-KIT/T670I **(m)** Imatinib-PDGFRα **(n)** Compound 14- PDGFRα **(o)** Compound 31- PDGFRα **(p)** D18-PDGFRα **(q)** D23-PDGFRα **(r)** D25-PDGFRα **(s)** D28-PDGFRα **(t)** D32-PDGFRα **(u)** D39-PDGFRα **(v)** D44-PDGFRα **(w)** D45-PDGFRα **(x)** Imatinib-PDGFRα/T674I **(y)** Compound 14-c-KITα/T670I **(z)** Compound 14- PDGFRα/T674I

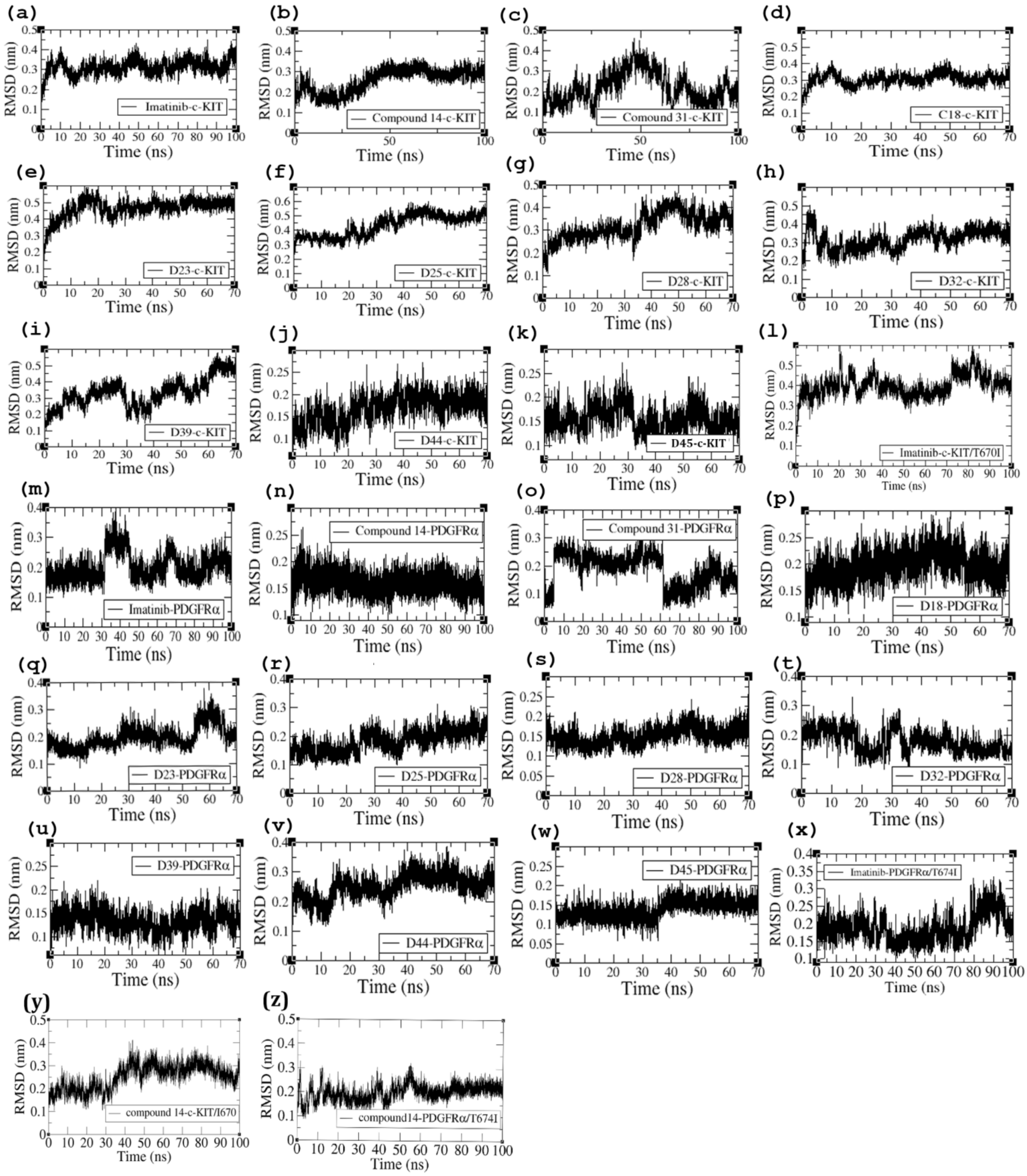

Figure S5. Hydrophobic surface of c-KIT and the binding interactions of compound 31 with the receptors. **(a)** The hydrophobic surface of the c-KIT binding site residues; Hydrophobic residues from the αC-helix and the catalytic loops are shown in stick representation **(b)** Binding interactions between **compound 31** and c-KIT. **(c)** Binding interactions between **compound 31** and PDGFRα.

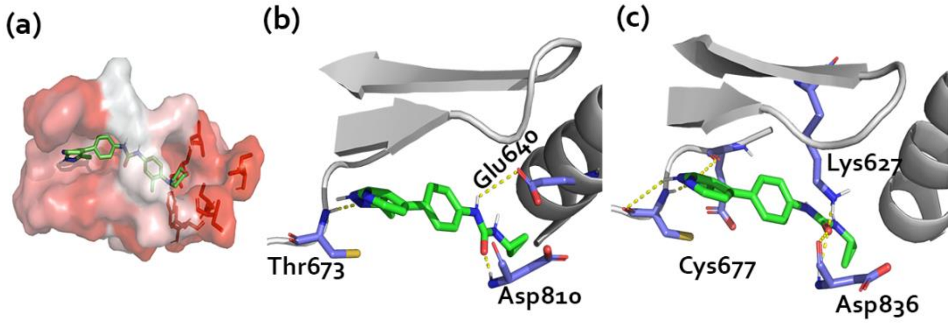

Supplement: Supplementary file 1 [file ijms-21-08232-s001.pdf]
